# Kinome-wide interaction modelling using alignment-based and alignment-independent approaches for kinase description and linear and non-linear data analysis techniques

**DOI:** 10.1186/1471-2105-11-339

**Published:** 2010-06-22

**Authors:** Maris Lapins, Jarl ES Wikberg

**Affiliations:** 1 Department of Pharmaceutical Pharmacology, Uppsala University, SE-751 24, Sweden

## Abstract

**Background:**

Protein kinases play crucial roles in cell growth, differentiation, and apoptosis. Abnormal function of protein kinases can lead to many serious diseases, such as cancer. Kinase inhibitors have potential for treatment of these diseases. However, current inhibitors interact with a broad variety of kinases and interfere with multiple vital cellular processes, which causes toxic effects. Bioinformatics approaches that can predict inhibitor-kinase interactions from the chemical properties of the inhibitors and the kinase macromolecules might aid in design of more selective therapeutic agents, that show better efficacy and lower toxicity.

**Results:**

We applied proteochemometric modelling to correlate the properties of 317 wild-type and mutated kinases and 38 inhibitors (12,046 inhibitor-kinase combinations) to the respective combination's interaction dissociation constant (K_d_). We compared six approaches for description of protein kinases and several linear and non-linear correlation methods. The best performing models encoded kinase sequences with amino acid physico-chemical z-scale descriptors and used support vector machines or partial least- squares projections to latent structures for the correlations. Modelling performance was estimated by double cross-validation. The best models showed high predictive ability; the squared correlation coefficient for new kinase-inhibitor pairs ranging P^2 ^= 0.67-0.73; for new kinases it ranged P^2^_kin _= 0.65-0.70. Models could also separate interacting from non-interacting inhibitor-kinase pairs with high sensitivity and specificity; the areas under the ROC curves ranging AUC = 0.92-0.93. We also investigated the relationship between the number of protein kinases in the dataset and the modelling results. Using only 10% of all data still a valid model was obtained with P^2 ^= 0.47, P^2^_kin _= 0.42 and AUC = 0.83.

**Conclusions:**

Our results strongly support the applicability of proteochemometrics for kinome-wide interaction modelling. Proteochemometrics might be used to speed-up identification and optimization of protein kinase targeted and multi-targeted inhibitors.

## Background

Protein kinases comprise a large family of membrane-bound and cytosolic enzymes, with 518 genes identified in the human genome [1]. All protein kinases catalyze the transfer of the γ-phosphate of adenosine triphosphate (ATP) to the hydroxyl group of tyrosine, serine, or threonine residues of protein substrates. Together with the protein phosphotases, kinases act as regulatory switches for essentially all cellular processes, including metabolic pathways, cell growth, differentiation, survival, and apoptosis. Abnormal function of protein kinases leads to development of many serious diseases, such as cancer, diabetes, inflammatory and autoimmune disorders, and diseases of the heart. In particular, many cancers (breast, ovary, lung, liver, colon, and prostate cancer, lymphoma, glioma, melanoma, and others) may be linked with increased activity of specific growth-factor-receptor tyrosine kinases due to overexpression, or mutations leading to constitutively active forms [2].

Great hopes were placed that inhibition of dysfunctional kinases will lead to new highly effective therapies. The first small-molecule kinase inhibitor, imatinib, was launched in 2001 as an anticancer agent for the treatment of chronic myeloid leukemia; its action being to inhibit the constitutively active form of Abelson tyrosine (ABL) kinase. Since then, eight compounds targeting the kinase catalytic domain were approved for treatment of various forms of cancer; over thirty kinase inhibitors are in the clinical phases of development, and many more are in preclinical pipelines.

A major problem in the development of kinase inhibitors is to achieve specificity. Most of the kinase inhibitors in current development interact with the kinases' ATP binding cleft, where they compete with ATP [3]. However, the ATP-binding site is highly conserved among all kinases and it is therefore difficult to design a drug selective for only one kinase at a time. Other functional domains that have been exploited to target kinases are also conserved among numerous kinases making the design of selective inhibitors problematic also in these cases. In fact, a large-scale screening undertaken by Fabian et al. [4] revealed that the three first FDA approved inhibitors actually interacted with about one sixth of the protein kinases included in the screen; each of them cross interacted with between 18 to 23 of 119 evaluated protein kinases. Seventeen other kinase inhibitors in pre-clinical and clinical phases of development were also tested in this study and were shown to possess various degree of promiscuity; only one of the compounds interacted with less than five kinases.

Many promising kinase inhibitors were abandoned early due to toxicity [5]. Yet another common reason for failure was lack of clinical efficacy. The latter problem can be attributed to the multitude and complexity of cellular signaling cascades, with redundant pathways and complex feed back mechanisms. Use of multi-targeted compounds that can selectively inhibit a specific group of kinases of such pathways might increase the chance to achieve clinical antitumor activity [6]. Yet another reason for lack of clinical efficacy is resistance that arises due to mutations in the targeted oncogene. *E.g*., drug resistance in imatinib-treated leukemia patients appears due to mutations in the BCR-ABL fusion protein. This prompts the need for new generations of drugs that can override the acquired resistance by inhibiting the mutated oncogene [7,8].

A computational method widely applied in drug design is quantitative structure-activity relationship (QSAR) modelling. QSAR models are used to optimize lead compounds for target activity and other properties (*e.g*., ADME and toxicity) and to perform virtual screening to find new hits. However, drawbacks of QSAR are that its models consider only properties of ligands and that it analyzes interactions with only one drug target at a time. Hence QSAR models are unable to generalize between multiple targets.

A more general approach is proteochemometric modelling, which we introduced some time ago to study differences in mechanisms of molecular recognition for groups of related proteins [9,10]. Proteochemometric models are based on experimentally determined interaction data for series of proteins interacting with series of ligands, like organic compounds, peptide inhibitors, substrates, etc. These data are correlated to descriptors of the two sets of interacting entities, which creates models that can be used to predict activities of yet untested ligand-protein combinations, as well as foresee activity profiles of novel unseen ligands and proteins.

Proteochemometric models take advantage of the fact that 3 D structures of homologous proteins are more conserved than their primary sequences and functions. Thus, proteins that have diverged functionally during evolution may still share the same structural organization and exploit similar molecular interaction mechanisms. The principle behind proteochemometrics is simple. It requires (1) consistent interaction data, (2) numerical descriptions of relevant physico-chemical and/or structural properties of both ligands and the protein macromolecules, and (3) a non-linear correlation method that jointly uses the two sets of descriptors to explain ligand-protein complementarities and interaction profiles. We have previously successfully applied proteochemometrics to create high-resolution models for ligand interactions with several classes of G-protein coupled receptors and for inhibition of multiple mutated variants of the HIV-1 protease. The aim of this study was to evaluate several types of kinase descriptors and compare the performance of different multivariate correlation methods in large-scale proteochemometric modelling of protein kinase-inhibitor interactions.

## Results

### Performance of different types of kinase descriptors in PCA and PLS-DA models

In order to compare the performance of the alignment-based approach and the five alignment-independent approaches used herein for describing protein kinase sequences we applied principal component analysis (PCA) and partial least-squares discriminant analysis (PLS-DA). PCA was performed to visualize how different types of descriptors separate the seven groups of protein kinases confined in the data set of 317 sequences. PLS-DA was used to obtain a quantitative measure of the ability of the descriptors to discriminate these groups. The seven kinase groups were as defined in [1], namely: AGC, CaMK, CK1, CMGC, STE, TK, and TKL. (The so-called atypical and other kinases were included in the PCA analysis but they were excluded from the PLS-DA modelling.)

**Figure 1:**
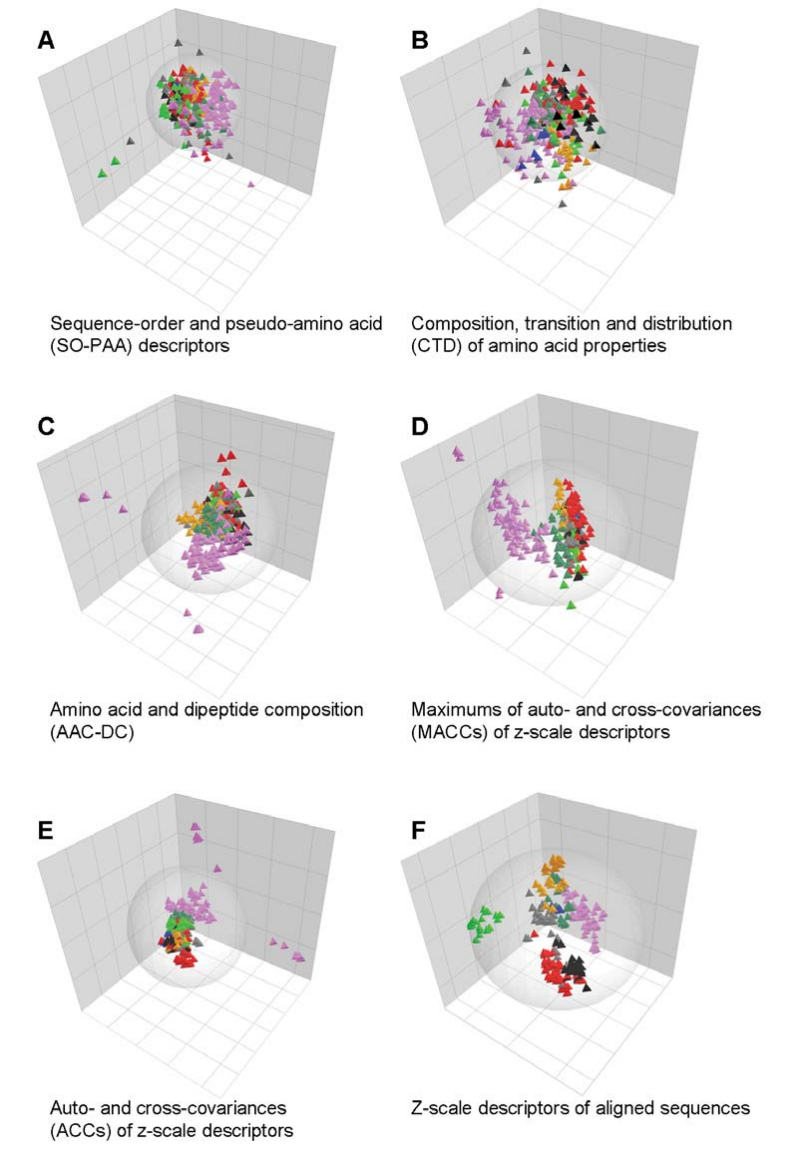
**Plots representing the separation of kinase groups in the three first components of PCA models**. Panels A-F show results of six PCA models using various types of alignment independent (A-G) and alignment based (F) kinase descriptions. Each one of the 317 protein kinases is represented by a tetrahedron, color-coded according to its belonging to a kinase group: black, AGC (named after member families PKA, PKG and PKC); red, CAMK (calcium/calmodulin regulated kinases); blue, CK1 (casein kinases); electric green, CMGC (named after member families CDK, MAPK, GSK3, and CLK); orange/amber, STE (homologues of yeast Sterile kinases); magenta, TK (tyrosine kinases); sea green, TKL (tyrosine kinase-like kinases); gray, atypical/other. Note that in Panels A and B kinase groups do not form distinct clusters, whereas in the other panels the largest kinase groups are clearly separated.

The first three principal components of the PCA models for the six sets of descriptors are visualized in Figure 1, Panels A to F. As seen from panels A and B, SO-PAA and CTD descriptors distribute the kinases in a more or less random fashion, albeit part of tyrosine kinases are separated from other groups, and the STE and CK1 groups are quite compact. Clustering into groups is more evident when the AAC-DC descriptors and MACCs of z-scale descriptors are used (Panels C and D). For these descriptors the location of the TK group, which is the largest group in the data set, shows almost no overlap with the other groups. Finally, the ACCs of z-scale descriptors (using the maximum lag *L *= 50; see below the reason for selecting this lag) and the z-scale descriptors of aligned sequences give good separation of most of the kinase groups (Panels E and F). However, a notable difference between the two last is that ACCs separate subgroups of TKs, while the first three PCs of descriptors of the aligned sequences do not reveal such sub-clustering. On the other hand, the alignment-based descriptors are the only ones that separate CMGC kinases as being substantially different from the other groups. As seen from Panel F, for the alignment-based approach the CMGC kinases form a distinct cluster in the first two PCs.

PLS-DA finds the directions in PC space where maximum separation among the classes is obtained and where each class forms a maximally compact cluster. In an ideal situation a cross-validated correlation coefficient Q^2 ^= 1 indicates that all members of a class are predicted to have **y **= 1, whereas all non-members are predicted to have **y **= 0. In reality Q^2 ^is always lower than 1, which is due to intra-class variations. Nevertheless, a Q^2 ^within the range 0.6-0.8 still indicates a good separation of classes, with few or no mispredictions. Should Q^2 ^drop down to 0.4-0.6, or even less, we have a warning that classes overlap and that the model will make multiple mispredictions. (Anyhow, the predictions would still be better than random. In fact, a random model has a Q^2 ^= 0).

**Figure 2:**
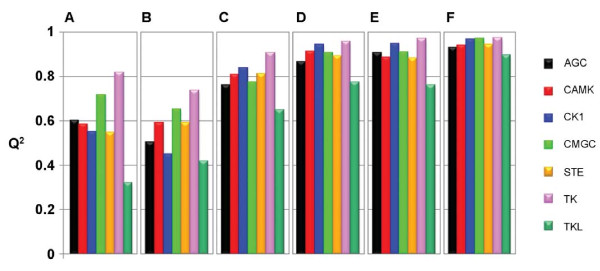
**Predictive ability of PLS discriminant analysis (PLS-DA) models estimated by five fold-cross-validation**. Kinase descriptions in the six models presented in panels A-F correspond to respective panels in Figure 1. Q^2 ^values may vary from 0 to 1. A low value indicates that a kinase group is randomly mixed with the others, while a value approaching 1 indicates a perfect discrimination. The overall high Q^2 ^values certify that the respective types of kinase descriptions have captured sequence properties that are present in members of a specific kinase group while being absent among all other kinases.

Cross validation results for each type of kinase description for each kinase group are shown graphically in Figure 2, where panels A to F present PLS-DA results for the same descriptor types as in Figure 1, A-F. Similarly as for the PCA models, z-scale based descriptions perform the best, with the alignment based approach performing over all the best. As seen, extremely high predictive ability was obtained with the Q^2 ^values for the seven kinase groups ranging from 0.89 to 0.97, the overall Q^2 ^being 0.94 (Figure 2, Panel F). Comparisons of all six panels of Figure 2 reveal that, irrespectively of the description type, the best separation is obtained for TKs. The lowest Q^2 ^values were for all descriptions obtained for TKL kinases suggesting that this group is more diverse than the other groups; (as its name indicates, the TKL group comprises enzymes that are phylogenetically related to TKs, although they are in fact serine-threonine kinases). However, cross-validation results showed that none of the TKL kinases was mispredicted as being non-member, and none of the other kinases was mispredicted as being member of TKL group in the models that used MACCs or alignment based descriptions. However, the model that exploited ACCs mispredicted one TKL kinase (the TNNI3K kinase).

### Selection of optimal lags for ACC and MACC transforms

An additional goal of the preliminary modelling was to identify the optimal complexity of the ACC and MACC descriptions. (In other words to find the maximum lag *L*, up to which descriptors contribute to improved separation of kinase groups). As described in Methods, covariances over long distances are less helpful in finding physico-chemical similarities in related protein sequences due to the differences in the length of segments that connect their functional units. Use of very many ACC or MACC terms with large lags may then give rise to chance correlations, deteriorating the resolution of any mathematical models created from them. By comparing PLS-DA models exploiting ACC and MACC descriptors with different maximum lags (*L *being 10, 25, 50, and 100) we showed that for both descriptor types the results were somewhat inferior for *L *= 10; the overall Q^2 ^being 0.76 and 0.86 for ACC and MACC based models, respectively. Increasing *L *to 25 gave major improvements (the overall Q^2 ^being 0.90 and 0.89, respectively); further increase to *L *= 50 produced yet slightly better models. Finally, including very long distance covariances with *L *= 100 led to slightly reduced predictive ability, the Q^2^s dropping to 0.88 and 0.87 for ACCs and MACCs, respectively. An interesting finding was that the performance of the two descriptor types was quite similar when the maximum lag was set to *L *= 25 and larger. This was so both in terms of overall Q^2^, and with respect to Q^2^'s for the seven groups of kinases (data not shown). Based on all these results we elected to use ACC and MACC descriptors with maximum lag 50 in all further modelling of kinase-inhibitor interactions.

### Performance of different types of kinase descriptors and multivariate correlation methods in predicting kinase-inhibitor activity

We used several machine learning methods to correlate the descriptors of kinase inhibitors and kinases to the interaction activities. The methods used were as follows: decision trees (DT), one nearest neighbour (1-NN) and k-nearest neighbour (k-NN) approach, support vector machines (SVM), and partial least-square projections to latent structures (PLS). The first four methods induce non-linear models, whereas PLS is a linear method. When using PLS we created both linear and non linear models; in the latter case the dataset included cross-terms derived from kinase and inhibitor descriptions.

The predictive abilities for new inhibitor-kinase combinations (P^2^) and new kinases (P^2^_kin_) as assessed by outer-loop cross-validation are presented in Table 1. The most predictive models were obtained using SVM, where for all three z-scale based description methods the P^2 ^values fell in the range 0.70-0.73 and the P^2^_kin _values in the range 0.67-0.70. The PLS (with cross-terms) and k-NN models performed almost as good. (However, the performance of the k-NN model exploiting ACC descriptions was inferior; its P^2^_kin _being only 0.53.) Models based on AAC-DC descriptors performed clearly worse than the z-scale based descriptions, but also here the SVM model was the most predictive; the P^2 ^being 0.68 and P^2^_kin _being 0.64, whereas the values of these parameters for PLS model were only 0.58 and 0.53.

**Table 1:** Results of proteochemometric modelling of kinase-inhibitor interactions using different types of kinase descriptions and different data analysis methods

Data analysis method:	DT		1-NN		k-NN		SVM		PLS		PLS (w/o cross-terms)	
**Kinase description:**	**P**^**2**^	**P**^**2**^_**kin**_	**P**^**2**^	**P**^**2**^_**kin**_	**P^2^**	**P**^**2**^_**kin**_	**P^2^**	**P**^**2**^_**kin**_	**P^2^**	**P**^**2**^_**kin**_	**P^2^**	**P**^**2**^_**kin**_
	
Composition, transition, and distribution (CTD) of amino acid properties	0.45	0.38	0.48	0.43	0.58	0.53	0.66	0.60	0.48	0.45	0.32	0.30
Sequence order and pseudo-amino acid (SO-PAA) descriptors	0.44	0.33	0.52	0.49	0.60	0.55	0.68	0.63	0.49	0.44	0.32	0.29
Amino acid and dipeptide composition (AAC-DC)	0.43	0.33	0.50	0.46	0.62	0.57	0.68	0.64	0.58	0.53	0.34	0.31
Maximums of auto- and cross-covariances (MACCs) of z-scales	0.46	0.30	0.55	0.55	0.63	0.63	0.70	0.67	0.66	0.63	0.35	0.32
Auto- and cross-covariances (ACCs) of z-scale descriptors	0.48	0.42	0.53	0.49	0.64	0.53	0.72	0.69	0.66	0.64	0.35	0.32
Z-scales of aligned sequences	0.49	0.43	0.55	0.58	0.65	0.64	0.73	0.70	0.67	0.65	0.34	0.32

Shown are the performances of proteochemometric models based on decision trees (DT), one nearest neighbour (1-NN) and k-nearest neighbour (k-NN) approaches, support vector machines (SVM), and partial least-square projections to latent structures, with (PLS) and without cross-terms (PLS w/o cross-terms). P^2 ^and P^2^_kin _indicate the squared correlation coefficient from outer loop of cross-validation for, respectively, new kinase-inhibitor combinations and new kinases.

The inferior performance for the AAC-DC descriptions is not surprising. In fact it seems quite unlikely that the fraction of any single dipeptide would show significant correlation with the functional properties of the kinases. Such correlations, however, can become evident for larger sets of dipeptide combinations (*i.e.*, tripeptides, tetrapeptides, and longer similar sequence stretches), giving an advantage to the SVM model which by the use of its non-linear kernel can approximate high-complexity interaction effects between the descriptors. The difference between the performances of SVM and PLS models is even larger when proteins are described by CTD or by SO-PAA descriptors; the P^2^_kin _for PLS models using these two sets of descriptors being, respectively, 0.45 and 0.44, compared to 0.60 and 0.63 for the SVM models.

For any set of descriptors the k-NN method outperformed 1-NN (see Table 1). However, the optimal number of neighbours found to be used by the cross-validation inner-loop was quite low, and ranged in all cases 3 to 5. The predictions of k-NN models are thus based on local subsets of the data set, and for this reason it would be problematic to use these models to draw any general conclusions on the molecular properties that determine kinase-inhibitor complementarity.

Finally, as expected, PLS modelling without use of kinase-inhibitor cross-terms explained only a minor part of the activity variation; the P^2^_kin _for all three z-scale-exploiting models being 0.32 (see Table 1). This result shows that the non-linear part which describes kinase-inhibitor selectivity dominate over the linear part that describes the average activity of a ligand for the protein series and the average activity of all ligands for a particular protein. The high non-linearity in the dataset is also likely the reason for the moderate success of the decision tree algorithm, which for any of the six used kinase descriptions created a massive tree with over 300 leaves explaining 65-71% of the activity variation (data not shown). However, all these trees suffered in ability to generalize to novel kinases; the P^2^_kin _for various descriptions ranging only 0.30-0.43.

### Distribution of prediction errors in SVM, PLS and k-NN models

**Figure 3:**
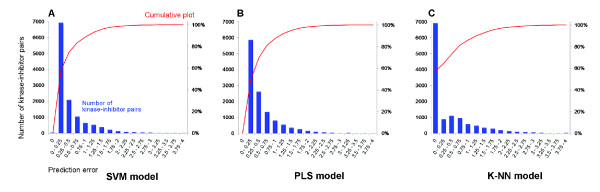
**Distribution of prediction errors in kinase-inhibitor interaction activity models**. Shown are prediction errors in models using three different data modelling methods, namely: support vector machines (Panel A), partial least-squares projections to latent structures (Panel B), and k-nearest neighbour approach (Panel C). Prediction errors are estimated by outer-loop cross-validation, iteratively excluding 1/5 of the kinases in the data set. The histograms represent the absolute values of prediction errors (*i.e*. blue bars; labelling on the left side of panels); the cumulative plot of prediction errors is represented by red lines; labelling on the right side of panels).

The performance of the SVM, PLS, and k-NN models exploiting z-scale descriptors of aligned sequences (*i.e*. the description that gave the best models) is further illustrated in Figure 3. The figure presents histograms for the prediction errors calculated in the outer-loop of cross-validation for 1/5 of the kinases that had been entirely excluded from the modelling (see Methods for details). The distributions of errors in the SVM and PLS models are very similar (*cf*. panels A and B). The cumulative plot demonstrates that in the SVM model the difference between predicted and observed pK_d _values range 0-0.25 logarithmic units for 57% of the kinase-inhibitor combinations; for 75% of the combinations they fall below 0.5 logarithmic units; for 89% they are less than one logarithmic units, and for 99% less than two logarithmic units. The corresponding fractions in the PLS model are 49%, 70%, 88%, and 98%. To interpret these results one should keep in mind that the total span of kinase-inhibitor activities exceeded five logarithmic units, namely from pK_d _= 5 to 10.62, and all non-interacting entities were assigned the numerical value pK_d _= 4; hence mispredictions by more than six units could be theoretically possible.

For the k-NN model the pattern of error distribution is quite different (Figure 3, Panel C). Here the prediction error was zero for more than one half of the non-interacting pairs (*i.e*. all their nearest neighbours had also been identified as non-interacting in the primary screen and were in the modelling assigned the same numerical value pK_d _= 4). However, 14% of the prediction errors exceed one logarithmic unit and 4% exceed two logarithmic units, thus indicating that predictions of the k NN model are less accurate compared to those obtained by SVM and PLS. In other words, activities for inhibitors interacting with overall quite similar kinases may vary a lot and regression models can better explain this than the nearest neighbour approach.

### Dependence of modelling performance on the size of the dataset

Although both SVM, PLS, and k-NN models showed good predictive ability they were based on more than 12,000 data points. It would thus be of obvious interest to know the robustness of the proteochemometric approach when less data are available. We therefore assessed the relationship between the sparseness of the data matrix used and the performance of the model. To this end we created models using 60, 40, 20, and 10 percent of all data. For example, when 10% of the data was used to calculate the P^2^_kin _value, the set of 317 kinases was randomly split into ten partitions of about equal size. Modelling was then performed using only one of these partitions at a time and the nine remaining partitions were used to evaluate the model obtained. The procedure of splitting the dataset was iterated ten times in order to assure reproducibility of the results. The P^2 ^and P^2^_kin _measures for models exploiting z-scale descriptors of aligned kinase sequences are presented in Table 2, where the values for 80% the dataset size are in fact identical with the above-presented results of 5-fold outer-loop cross-validation (*cf*. Table 1). The performance of the models decreases only slightly when 40-60% of the whole dataset is used for the model building, and the models are still predictive when as few as 10% of all kinase-inhibitor combinations or when 10% of all kinases are present in the dataset (*i.e*., estimating P^2 ^and P^2^_kin_, respectively). Moreover, the small margins between the P^2 ^and P^2^_kin _parameters indicate that the reliability of predictions for "new unassayed kinases" does not differ much from the reliability of predictions for the kinases for which some interaction data have been already assayed and used in the modelling. Comparisons of the results for the three data analysis methods also indicate that their performance is more similar for larger datasets. For sparsely populated datasets the performance of k-NN method deteriorates faster than for the SVM and PLS methods.

**Table 2:** Results of k-NN, SVM, and PLS modelling using subsets of full kinase-inhibitor dataset

Data analysis method:	k-NN		SVM		PLS	
**Size of the dataset:**	**P^2^**	**P**^**2**^_**kin**_	**P**^**2**^	**P**^**2**^_**kin**_	**P^2^**	**P**^**2**^_**kin**_
	
80%	0.65	0.64	0.73	0.70	0.67	0.65
60%	0.60	0.59	0.70	0.67	0.64	0.62
40%	0.52	0.51	0.65	0.62	0.58	0.56
20%	0.44	0.43	0. 56	0.53	0.49	0.47
10%	0.32	0.30	0.47	0.42	0.41	0.37


For explanation of abbreviations see legend to Table 1.

### Predicting interacting versus non-interacting kinase-inhibitor pairs

Although all models predict interaction activities on a continuous scale, they can also be used to predict whether new inhibitors and kinases interact or not. In the quantitative modelling we assigned the value pK_d _= 4 to all inhibitor-kinase combinations that had been found not to interact in the primary screen - the screen for which the detection limit was pK_d _= 5. Hence if the activity predicted for an inhibitor-kinase pair falls below a pre-specified threshold level, the pair could be classified as non-interacting, while if it falls above this threshold it could be classified as interacting. The selection of the threshold value will affect the sensitivity and specificity of the classification, which can be defined as:

A common measure for the classification quality is the Receiver Operating Characteristic (ROC) curve, which is plotted as sensitivity versus one minus specificity upon varying the discrimination threshold value. The area under the ROC curve (AUC) is a measure of the discriminatory power of a classifier, which is insensitive to class distributions and the costs of misclassifications; AUC = 1 indicates perfect classification, while AUC = 0.5 means that the classifier does not perform better then random guessing.

**Figure 4:**
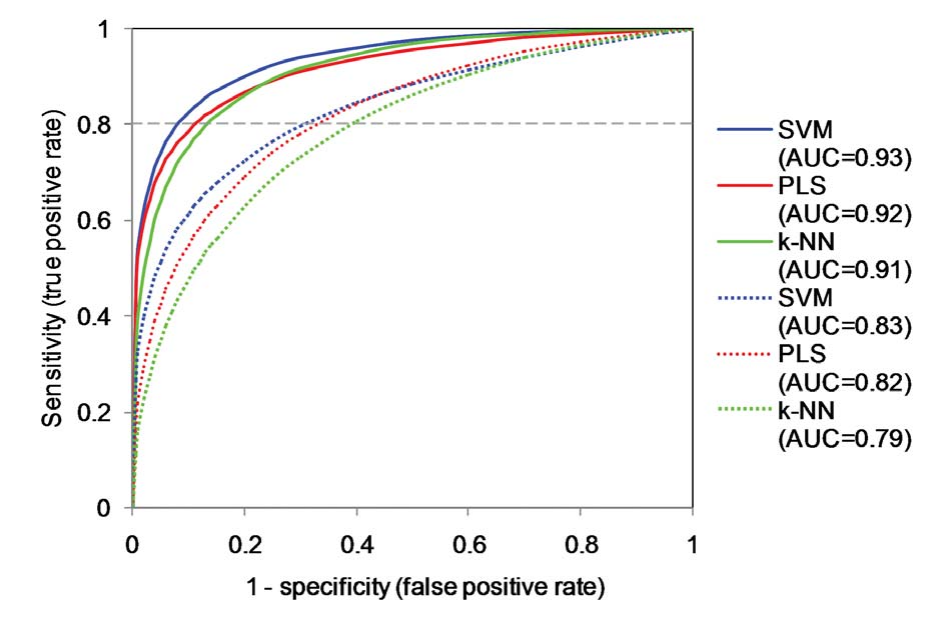
**ROC curves for SVM, PLS, and k-NN models**. Shown are ROC curves for SVM, PLS, and k-NN models built on data for 80% (solid lines) and for 10% (dashed lines) of 317 protein kinases. The area under the ROC curve (AUC) is a measure of the discriminatory power of a classifier.

Figure 4 compares ROC curves for the k-NN, SVM, and PLS models, built on the largest and on the smallest sets of kinases as described in the previous section (*i.e*. using 80% and 10% of all 317 kinases). Inspection of Figure 4 shows, for instance, that at a sensitivity of 0.80 the SVM model build on the largest set of kinases has a specificity of 0.92. In other words, using a threshold that identifies 80% of truly active kinase-inhibitor pairs as being active, the number of false-positives amounts to only 8%. The performance of the PLS and k-NN models were slightly worse, at the sensitivity of 0.80 the false-positives amount to 11 and 13%, respectively.

The good performance of the classification is further indicated from the ROC areas, which for the models built on 80% of the kinases were 0.93, 0.92 and 0.91 for, respectively, the SVM, PLS, and k NN model. Interestingly, the models built on only 10% of the kinases also show good classification performance, the ROC areas being, respectively, 0.83, 0.82, and 0.79 for SVM, PLS, and k-NN models. This finding indicates that even in the cases when quantitative models do not possess very high predictive ability in terms of P^2^, they may still be able to separate active and inactive kinase-inhibitor combinations. Accordingly, our models should be useful for virtual large-scale screening to select the promising objects prior to their experimental testing, while sorting away objects with a less probability of having the properties sought for in a development project.

## Discussion

Design of selective and multiselective medications requires understanding of the properties of the biological targets that distinguish the chosen target(s) from numerous similar "anti-targets" encoded in the human genome. Contemporary drug design has to a large extent been focused to structure-based methods where ligands are designed to fit into a binding pocket of the target. This requires knowledge of the exact 3 D structures of the targets and anti-targets, which is a problem for protein-kinases as X-ray structures have been solved for only 124 human protein kinase domains [11].

Proteochemometrics, on the other hand, has a distinct advantage when the studied proteins share the same structural organization since primary amino acid sequences can then be used without the need to have high-resolution 3 D structures of the targets. Proteochemometrics has also the advantage that multiple targets and anti-targets can be encompassed in one single model. Structural alignments of protein kinases have shown that they all contain universal conserved subdomains whereas their amino acid sequences still show quite notable variation. In fact, there is generally a much higher degree of conservation of the 3D-structures among protein families than of their primary sequences [12]. The average pair-wise sequence identity over the kinase domains falls below 30%, and only a small fraction of residues are markedly conserved across the entire superfamily [13]. Use of sequence-derived descriptions can hence be considered to be a rational approach for kinase representation in multivariate modelling, stated that the sequence descriptions are made in such a way that they are relevant for the structural and functional organization of the kinases. Descriptions can be derived based on prior sequence alignments or in alignment independent ways, the latter approaches are advantageous for less similar sequences, when unambiguous alignments are impossible to obtain.

In the first phase of this study we performed PCA and PLS-DA, using one set of alignment based and five sets of alignment independent descriptors of protein kinase amino acid sequences. The purpose of this analysis was to evaluate the ability of the different descriptions to separate kinases into groups according to their functions. PLS-DA for the best model (which exploited alignment based z-scale descriptions) afforded excellent separation of the seven groups of kinases; the cross-validated squared correlation coefficients fell between 0.93-0.98 for six of the groups, while for the more diverse tyrosine kinase-like kinase group it was 0.89.

As explained in the Methods section, PLS-DA models create regression equations for each of the modelled classes and thus identify properties that are more typical, or even unique, for a particular class compared to the other classes. Thus, inspection of the alignment based PLS-DA regression equation exploiting z-scale descriptors reveals that in some cases the description of the physico-chemical properties of very short sequence stretches and even of single residues are sufficient to separate all members of one kinase group from all other kinases. In one such example, when we inspected the alignment based PLS-DA model we revealed that a conserved proline residue located surrounded by two hydrophobic amino acids in the activation loop of the TKs sequences is the sufficient pattern for class separation. In the majority of the cases this triplet is embraced by two positively charged lysine or arginine residues (*e.g*., the sequence stretch being KFPIK in ABL1 kinase, KVPIK in EGFR kinase, and RLPVK in KIT kinase). Analysis of the alignment independent PLS-DA model exploiting AAC-DC descriptors further identifies that groups of kinases are often distinguished by the model by small sets of dipeptides (for instance, each of dipeptides CW, VW, RN, and GM is present in more than 90% of TKs compared to only 15-35% of kinases from the six other groups). Such identified specific sequence residues or patterns, which may be identified by our models, could accordingly potentially be addressed in the design of targeted and multi-targeted drugs. In fact, a few such amino acids (sometimes termed 'selectivity filters') have been previously exploited in drug design for kinases. This includes the so-called gatekeeper residue, which is a bulky amino acid present in most kinases, while 20% of the kinases have a threonine at this position. The property was used in design of selectivity for ABL kinase inhibitors. (However, unfortunately, the residue position is also a common site for mutations that confer resistance to imatinib, gefitinib, and erlotinib [14]). A study of Cohen et al. [15] designed inhibitors for RSK family kinases by targeting two selectivity filters in the ATP binding site, namely the threonine gatekeeper and a cysteine residue, which is an uncommon amino acid in the kinases' active site. These two amino acids that distinguishes RSKs from other protein kinases were sufficient to confer high activity of the designed inhibitor.

Although we here limited PLS-DA modelling to separation of seven major groups of the kinase superfamily the analysis can be performed hierarchically at any resolution, *e.g*., to delineate particular families, subfamilies, and even single kinases.

In the subsequent studies we created quantitative models for kinase-inhibitor interaction activities using the six types of kinase descriptions and performing correlations using SVM, PLS, k-NN, and decision trees. The small molecule inhibitors were in all models represented by a unified set of 3D-structural and physicochemical property descriptors. Models that exploited z-scale descriptions of the alignable parts of the protein kinase sequences performed the best. However, using ACC or MACC transformations gave only slightly inferior models when correlations to the activity data were done by SVM or PLS. ACC transformed descriptors performed worse with the k-NN approach, while MACC transformations resulted in a weaker model with use of decision trees. The advantages of ACC and MACC transforms are that they do not require prior alignment and that they are calculated from full-length sequences of kinase domains, which in the present data set varied from 194 to 606 residues (albeit for about one half of kinases it ranged 240-260 residues; for less than 30% kinases it exceeded 280 residues). Whereas ACCs reflect the covariances of amino acid properties over whole sequences, MACCs pinpoint individual pairs of residues with specific property combinations. MACC based models may thus identify patterns that are not confined to the same location in each and every protein and/or are situated in sequence stretches that can not be aligned unambiguously over the whole dataset. Consequently, models exploiting MACCs may complement the alignment-based models in analysis and prediction of kinase-inhibitor interactions. The three other descriptions for the protein sequences used (CTD, SO-PAA, and AAC-DC) showed inferior performances compared to z-scale based descriptions and thus appear less useful in proteochemometric modelling.

SVM outperformed the other data analysis methods, including PLS, in both the prediction accuracy for the active kinase-inhibitor combinations as manifested by P^2 ^and P^2^_kin _parameters (Tables 1 and 2) and in the ability to distinguish interacting versus non-interacting kinase-inhibitor pairs as revealed by the areas under the ROC curves (Figure 4). Accordingly, SVM seems to be the optimal choice for predicting full kinome-wide selectivity profiles of the existing compounds, and for virtual screening to find new hits with desired selectivities. However, an important point is that SVM is essentially a 'black box' technique, which makes interpretations of its models difficult. Thus, even if the performance of SVM in virtual screening is superior to PLS, it is problematic to comprehend which of the molecular properties of kinases and inhibitors that are important in the model. PLS contrasts to 'black box' methods like SVM and to locally derived kNN and DT models because it expresses the correlation results in a single straightforwardly interpretable regression equation. Moreover, PLS provides additional tools for model diagnostics, such as score and loading plots and 'distance to model' parameters that allow identification of outliers and assessment of reliability of extrapolations outside the modelled chemical and interaction spaces [16]. Consequently, the parallel use of PLS and SVM modelling techniques may be advantageous when one aims at obtaining models for both predictions and interpretations, and cross-checking of model performances. (In this context it ought to be mentioned that several approaches have been recently suggested to give SVM models some transparency [17-19], which may be in the advantage for use of SVM in proteochemometric modelling).

The models built on small sub-parts of the dataset showed the robustness of the proteochemometric modelling approach. Thus, even for the smallest dataset comprising only about 30 kinases the SVM and PLS models showed acceptable predictive ability. The performances of the models based on small data-sets were even more impressive in prediction of interacting versus non-interacting kinase-inhibitor pairs; the discriminatory power of SVM and PLS models being, respectively, 0.83 and 0.82 for the models created on 30 kinases (compared to 0.93 and 0.92 for the largest dataset size). These results may have a wide impact to the protein kinase field as they mean that a relatively limited amount of experimental work is needed to afford qualitative and quantitative interaction models that will generalize for the whole kinome.

Success of any empirical modelling depends on the quality of data, which in proteochemometrics should comprise accurate activity measurements and descriptions of relevant physico-chemical and/or structural properties of proteins and their ligands. Yet another prerequisite for proteochemometrics is an adequate composition of the dataset, which should be balanced and include both interacting and non-interacting protein-ligand combinations. Unfortunately, 'negative' results are often omitted in study reports. Moreover, interaction databases populated by data from multiple series, contain typically activities for a fairly low fraction of all possible ligand-protein combinations, which implies that a bulk of the non-interacting entity pairs are absent. Modelling of sparse data matrices with overrepresented high activity data would inevitably give rise to false-positive predictions. Hence, the success of any modelling study owes most to using a well-balanced dataset, such as the here used dataset comprising data for both active and inactive kinase-inhibitor combinations for more than one half of the human kinome.

Although the modelled dataset covered more than 12,000 interactions, the series of 38 kinase inhibitors can not be considered as large, even though it included seven of the eight presently approved anticancer agents as well as other compounds with mutually dissimilar inhibition profiles. One can thus expect to gain further improvements by analyzing data for many more chemical compounds providing wider and denser coverage of the chemical and interaction spaces. In the present study the dataset parts for modelling and validation were selected randomly to assure objective assessment of the modelling performances. However, it is possible to apply statistical experimental design [20] to choose small representative panels of kinases to be used for assaying and interaction modelling. One technique is D-optimal design that could be used to select kinases that cover most of the diversity of the kinase sequence and activity space. Designed molecular libraries have proven much more informative than random collections, and they have been shown in some cases to allow a 10^3^-10^4 ^fold reduction of the experimental work required, while still retaining the full generalization ability of derived interaction models [21,21]. We can hence conclude that the values in Table 1 are the lowest limits of the predictive abilities, which would be surpassed in any models for datasets of the same size if kinases were selected according to principles of statistical experimental design. Hence, for any experimental work to be undertaken in the kinase field following this study we would strongly encourage the use of experimental design. The final outcome will be kinome wide models that can predict the interaction strength of a random chemical over all known protein kinases.

## Conclusions

In this study we developed kinome-wide proteochemometric models for the prediction of kinase-inhibitor interaction profiles. We compared several alignment-based and alignment-independent approaches for the description of protein kinases, evaluated the performances of linear and non-linear correlation methods, and investigated the relationship between the size of the dataset and the predictive ability of the models obtained. Our best models are highly predictive on a quantitative scale, and can delineate interacting and non-interacting kinase-inhibitor combinations. One of the findings of this study is that models built on quite limited amount of kinase data are still capable to generalize over the whole human kinome. We thus foresee that the here shown routes to concomitant proteochemometric kinome wide modelling will markedly speed-up the discovery and optimization of protein kinase targeted and multi-targeted drugs.

## Methods

### Interaction activity data

We used the dataset published by Karaman et al. [23] comprising dissociation constants (K_d_) of 38 small-molecule kinase inhibitors tested against a panel of 317 human kinases, in total 38 × 317 = 12,046 activities. All major kinase groups, as defined by Manning et al. [1], were represented in the dataset, namely: AGC, CaMK, CK1, CMGC, STE, TK, and TKL. The kinase inhibitor series included approved drugs (dasatinib, erlotinib, gefitinib, imatinib, lapatinib, sorafenib, and sunitinib), trial drugs and experimental compounds (flavopiridol, roscovitine, and others), and the natural product staurosporine. For 24.8% of the inhibitor-kinase combinations an activity better than 10 μM had been observed in a primary screen, and the exact K_d _values were then determined. The dissociation constants found ranged from 10^-5^M to 2.4 × 10^-11^M and were expressed as negative logarithms of the K_d _values (pK_d_); the transformed values ranging from 5 to 10.62. In order to obtain a full data matrix we assigned a numerical value pK_d _= 4 to the inhibitor-kinase pairs that had been identified as not interacting in the primary screen; *i.e*., pK_d _was set one unit lower than the threshold value (pK_d _= 5) of the primary screen. This was a trade-off between two qualities of the conceived mathematical models to be derived from the data: a very high margin would prioritize discrimination between the active and inactive kinase-inhibitor pairs on the expense of the accuracy for the predictions for the active ones; on the other hand, a low margin would reduce the model's discriminative ability between interacting and non-interacting pairs. Our selected value seemed reasonable since it would allow achieving both goals, stated that the errors of prediction of a model do not exceed one logarithmic unit.

### Description of kinase inhibitors

The structures of kinase inhibitors were drawn by ISIS/Draw and converted to 3 D by the Corina unit of the Tsar 3.3 (Accelrys, Inc.) software. Partial atomic charges were derived using the Charge2 utility and the geometries were optimized by energy minimization using the Cosmic utility of Tsar 3.3. Compounds were then characterized by various molecular descriptors using Dragon 2.1 software (Talete S.r.l.). The following descriptor classes were calculated: constitutional descriptors, counts of functional groups and atom-centered fragments, geometrical descriptors, charge and aromaticity indices, empirical descriptors, and molecular properties. When two descriptors were highly correlated (pairwise r^2 ^> 0.9), we excluded the one showing the highest correlation with any other descriptor of the descriptor set. In this way, 150 molecular descriptors were obtained for each inhibitor for the modelling. All descriptors were mean centred and scaled to unit variance prior to use in modelling.

### Description of protein kinases

The panel of protein kinases comprised 317 entities (*i.e*., more than a half the known human kinome). Of these 28 contained point or cassette mutations, and a few kinases contained deletions of up to eight residue long sequence stretches. The sequences for the kinases' kinase domains were retrieved from KinBase database http://kinase.com/kinbase. Although the length of the kinase domains varied from 194 to 606 amino acids, almost 90% of them were just between 240 to 300 amino acids long.

#### Alignment-based physico-chemical z-scale description of kinase sequences

We used two types of kinase sequence descriptions: alignment based and alignment independent. For the alignment based, a multiple sequence alignment was performed over the entire sequence set by the ClustalW 2.0 software [24], using its default settings (GONNET 250 matrix) and applying ten iteration cycles to refine the progressive alignment. Those parts of the alignment that contained gaps for more than 50% of the kinase sequences were removed from the alignment, which left 264 aligned positions. (These gaps corresponded to sequence stretches that were quite unique among most kinases and they were located far from the ATP binding site). The aligned positions were then described by amino acid physico-chemical properties encapsulated in the five z-scales, z_1_-z_5_, derived by Sandberg et al. [25]. Z-scales are quantitative descriptors obtained from principal component analysis (*vide infra*) of 26 measured and computed physico-chemical properties of the 20 naturally encoded amino acids and 67 synthetic alpha amino acids. The three first of these z-scales describe about 70% of the variation in the original data, and all five describe more than 95% of the variation. Being principal components, z-scales are mean-centered and uncorrelated to each-other, and can be tentatively interpreted as reflecting hydrophobicity (z_1_), steric properties (z_2_), polarity (z_3_) and other electronic properties (z_4_, z_5_) of amino acids. In this way, the differences in physico-chemical properties of the aligned kinase sequences were represented by 264 × 5 = 1320 descriptors.

#### Auto- and cross-covariances (ACCs) of z-scale descriptors

Z-scales are directly useful for encoding proteins stated that the proteins show substantial conservation in their 3 D structural organization and that their primary sequences are conserved to the extent that alignments can be done unambiguously. However, if sequences are aligned wrongly our attempts to find similarities and differences in the proteins' physico-chemical space would be thwarted. Therefore, methods have been sought to avoid the alignment step and transform sequence descriptions directly into uniform matrices. One such method, the auto- and cross-covariance (ACC) transform, describes changes in some property or some property combinations over sequence stretches of different lengths [26]. This is done according to the equations:

where AC represents auto-covariances of the same property (z-scale) and CC the cross-covariances of different z-scales, and where *z *= 1, 2, ..., *Z *(*Z *is 5, *i.e*. the number of z-scales), *i *= 1, 2, ..., *N-lag *(*i *is the amino acid position in the sequence and *N *the total number of amino acids), *lag *= 1, 2, ..., *L *(*L *is the maximum lag, *i.e*. the longest sequence stretch used, which can be up to the length of the shortest sequence in the dataset), and *V *is the z-scale value. The total number of ACC terms depends on the chosen *L *and on the number of z-scales, and is *L *× *Z*^2^.

Larger maximum lags *L *allow for more detailed description accounting for interactions of amino acids at distant parts in a sequence. However, even closely related proteins differ often by sequence insertions/deletions. As a result, the probability of assigning an interaction to the same ACC term is inversely proportional to the distance between the sequence positions. Long distance covariances would hence be less helpful in finding physico-chemical similarities in related sequences. We here calculated ACCs with maximum lags 10, 25, 50, and 100.

#### Maximums of auto- and cross-covariances (MACCs) of z-scale descriptors

ACC-transformations provide a uniform set of descriptors that are independent of the length of each sequence and which are able to capture characteristic physico-chemical patterns of the protein. One limitation of ACCs is that specific local sequence patterns may become concealed by the overall properties of the given sequence. Another drawback is the difficulties to make interpretations. For example auto-covariances of the z_1_-scale would be similar for a sequence consisting of predominantly hydrophilic amino acids (represented by positive values) and a sequence consisting of predominantly hydrophobic amino acids (negative values). In both cases multiplications give positive values. (AC_z1 _terms would, however, separate such two sequences from sequences where hydrophilic amino acids alternate with hydrophobic ones with certain periodicities).

To cope with these limitations of ACCs, a modified algorithm was suggested in [27], where the positive and negative descriptor values are considered separately and only the maximum values for all possible interactions at each lag is used to describe the sequences. Of the two algorithms developed in [27] we applied the MACC1 transformation giving *4 *× *L *× *Z*^2 ^terms; *i.e*. four times as many descriptors as an ACC with the same maximum lag. (The alternative MACC2 algorithm was not used as it ignores the direction of a sequence and hence seemed inappropriate for encoding proteins). We here calculated MACCs with maximum lags 10, 25, 50, and 100.

It may be pointed out that, whereas each ACC term is calculated from the whole protein sequence, the corresponding four MACC1 terms represent extremes of particular physico-chemical property combinations somewhere in the sequence. The MACC descriptions thus retain full interpretability and can be traced back to each residue pair. However, they may overstate the roles of extreme physico-chemical properties for a protein structure/function and depreciate the roles of 'moderate' amino acids.

#### Composition, transition and distribution (CTD) of amino acid properties

The CTD alignment-independent descriptors were proposed by Dubchak and coworkers [28], and are based on seven amino acid properties (attributes): 1) hydrophobicity, 2) normalized van der Waals volume, 3) polarity, 4) polarizability, 5) charge, 6) secondary structure, and 7) solvent accessibility. For each of these seven attributes, amino acids are divided into three classes. *E.g*., for the hydrophobicity attribute, class 1 comprises polar amino acids (RKEDQN), class 2 neutral amino acids (GASTPHY), and class 3 hydrophobic amino acids (CLVIMFW). The *composition *descriptors then represent the overall percentage of each class in the sequence. Since there are seven attributes and three classes, 7 × 3 = 21 composition descriptions can be computed. The *transition *descriptors represent frequencies with which an attribute changes class along the sequence, *e.g*., a class 1 amino acid is followed by a class 2 amino acid or *vice versa*. Since there are three possible transitions between classes, 7 × 3 = 21 transition descriptors can be computed. The *distribution *descriptors represent the distribution of each attribute in the sequence. For each attribute and for each class, five distribution descriptors are computed based on the following criteria: location of the first residue, 25% residues, 50% residues, 75% residues and 100% residues with a given property. For instance, if the total length of a sequence is *N *amino acids, and all polar amino acids (*i.e*. members of hydrophobicity class 1) are among the first *i *residues of the sequence, then the distribution descriptor for 100% residues of the given class would be calculated as *i*/*N*. Thus, the total number of distribution descriptors is 5 × 7 × 3 = 121. CTD descriptors were computed by using PROFEAT (Protein Feature) web server [29].

#### Sequence-order and pseudo-amino acid (SO-PAA) descriptors

The sequence-order and pseudo-amino acid descriptors were proposed by Chou [30,31] and are used most successfully to predict protein subcellular location. We here used the PROFEAT web server to calculate 60 sequence-order-coupling numbers, 100 quasi-sequence-order descriptors, and 50 pseudo-amino acid descriptors. The sequence-order-coupling numbers are derived from the physico-chemical distance matrix between pairs of amino acids. The coupling number of rank *d *is defined as the sum of squared physico-chemical distances between all amino acids being located *d *residues from each other. This is mathematically described by the equation:

where *d*_*i,i+d *_is the physicochemical distance between the two amino acids at position *i *and *i+d*, and *N *is the total length of the sequence. PROFEAT allows computing these descriptors starting from rank *d *= 1 (*i.e*. neighbouring residues) up to *d *= 30 and using two different distance matrices (physico-chemical distance by Schneider-Wrede and chemical distance by Grantham) [29].

Quasi-sequence-order descriptors are thereafter computed from coupling numbers and from protein amino acid composition (see [29] for mathematical equations). Fifty quasi-sequence-order descriptors can be derived from each set of coupling numbers. The first 20 quasi-sequence-order descriptors reflect the effects of the amino acid composition and are calculated according to the equation:

where *a *is one of the twenty natural amino acids, *f*_*a *_is the normalized occurrence for this amino acid, and *w *is a weighting factor (*w *= 0.1).

The thirty other quasi-sequence-order descriptors reflect the effects of sequence order, and are defined as:

where the rank *d *is from 1 to 30.

Fifty pseudo amino acid descriptors were computed similarly as quasi-sequence order descriptors. However, the coupling numbers in the equations were replaced by more complex correlation factors reflecting various physico-chemical properties of amino acids (see [31] for details). The whole set of SO-PAA descriptors thus comprised 210 alignment independent descriptors encapsulating both the quantitative (physicochemical) and qualitative (amino acid letter code) sequence properties.

#### Amino acid and dipeptide composition (AAC-DC)

Amino acid composition descriptors represent the fractions for each of the twenty natural amino acids in a protein sequence, while dipeptide composition descriptors represent the fractions of 20 × 20 = 400 possible dipeptides in the sequence [32]. Despite its simplicity the method has been applied successfully, *e.g*., for classification of G-protein coupled receptors [33,34], nuclear receptors [35], predictions of protein fold and predicting the subcellular localization of proteins [36-38]. Amino acid and dipeptide composition descriptors were computed by using the PROFEAT server.

### Data preprocessing

All descriptors were mean-centered and scaled to unit variance prior to their use. In order to account for differences in the number of inhibitor and kinase descriptors, block scaling was applied. This was done by assigning each block the weight 1/sqrt(N), where N is number of descriptors in the block. In this way, the total sum of variances of all descriptors in each block became equal to 1. The response variable (pK_d_) was mean centered prior to applying data analysis.

### Data analysis

#### Principal component analysis (PCA)

PCA is a multivariate projection method, which provides compression of datasets containing large numbers of variables (see [39] for algorithms and geometrical interpretation). Contrary to the original variables, which are always multicollinear, the so-called principal components (PCs) are orthogonal to each other; the first component extracts the largest variance in the dataset, the second component extracts the largest of the remaining variance, and so on. The major patterns within the original data can often be captured by a small number of components.

All the variance in a dataset with *N *objects is explained by *N*-1 or less PCs. Thus, all descriptors of kinase inhibitors in the present dataset could be transformed into 37 PCs without any loss of information, and with the preservation of full interpretability. Similarly, any number of descriptors of 317 kinases can be compressed to 316 PCs (in fact, already half of this number explained over 90-95% of the variance in any of the six sets of kinase descriptions used herein).

#### Partial least-squares projections to latent structures (PLS)

PLS can be considered as an extension of PCA, which along with the independent variables (**X **matrix) deals with one or several dependent variables (**Y **vector or matrix). PLS aims to find the relationship between the two matrices and to develop a predictive model. This is achieved by simultaneously projecting **X **and **Y **to latent variables (PLS components), with an additional constraint to correlate them. (Thus, compared to PCs, the PLS components are tilted to maximize covariance between projections of **X **and **Y**). PLS derives a regression equation for each **y **variable where the regression coefficients reveal the direction and magnitude of the influence of **X**-variables on **y **[16].

A special case of PLS is PLS discriminant analysis (PLS-DA) where **y **variables are categorical and express the class membership of objects (members of a given class are numerically represented by the value 1 while non-members are represented by 0).

Several algorithms have been developed for performing PLS; here we used orthogonalized-PLS [40] as implemented in Simca-P+ 11.5 (Umetrics AB) and NIPALS [41] as implemented in Unscrambler-9.8 (CAMO Software AS) (the latter algorithm was applied for PLS-DA modelling). An important decision in PLS is the choice of the number of PLS components. Each extracted component increases the explained variation of both **X **and **Y**. However, while the first components normally find real correlations between the two blocks, increased model complexity may give rise to chance correlations. To avoid overfitting we applied five-fold inner-loop cross validation (see below).

#### Accounting for non-linear cooperative effects in PLS modelling

PLS is a linear correlation method. However, in proteochemometrics there is a need to describe non-linear ligand-protein interaction effects (*i.e*., those effects that are governed by the complementarity of the interacting moieties and determine the selectivities for the interactions) [9]. This is typically done by deriving cross-terms between ligand and protein descriptors. Since the number of cross-terms is equal to the product of ligand and protein descriptors it may be unfeasible to calculate them directly. *E.g.*, having at hand 150 inhibitor and 1,320 z-scale descriptors, computing cross-terms would result in 198,000 new variables, which would make any further analysis highly resource consuming. A practical approach is rather to compute the cross-terms from the principal components of the original descriptors. For calculation of cross-terms we here used all 37 PCs of the ligand descriptors, but only as many of PCs of kinase descriptors that explained 95% of their total variance (this allowed us to further reduce the size of the datasets by a factor of two). Cross-terms were scaled to Pareto variance; the block weight for cross-terms was initially set to 0 and thereafter increased by a regular step size until an optimal PLS model (according to inner loop cross-validation) was obtained. We have earlier shown that this approach exerts no negative influence on the final modelling results [42].

#### Support vector machines (SVM)

SVM is a machine learning technique for classification and regression that uses linear or non-linear kernel-functions to project the data into a high-dimensional feature space. Correlation is then performed in this hyperspace based on the structural risk minimization principle; *i.e*., aiming to increase the generalization ability of a model [43,44]. We induced non-linear proteochemometric regression models using the epsilon-SVR method and radial basis function kernel as implemented in the libSVM 2.88 software [45]. Five-fold inner-loop cross validation was performed to find optimal values for the width of the kernel function γ and error penalty parameter C.

#### K-nearest neighbour method (k-NN)

The k-NN algorithm predicts **y **values for a test set object as the average (or weighted average) of the **y **values of its *k *nearest neighbours in the training-set. k-NN models were induced using the Weka 3.6 software [46]. We characterized the similarity between inhibitor-kinase pairs from the Euclidian distance in the **X **descriptor space and applied 1/distance weighting, as described [47]. In contrast to PLS and SVM modelling, where the inhibitor and kinase descriptor blocks were scaled to equal total variance, the relative scaling of the descriptor blocks was varied systematically in the k-NN modelling by multiplying the block weight for kinase descriptors by factors 0.25, 0.5, 1, 2, and 4; (in this way, kinase descriptors obtained lower or higher importance than inhibitor descriptors in assessing inhibitor-kinase complex similarity). Five-fold inner-loop cross validation was applied to find the optimal scaling and number of nearest neighbours for prediction.

#### Decision trees

Decision trees were created using the M5P algorithm [48] as implemented in Weka 3.6. This algorithm derives linear regression models at the terminal nodes (leaves) of the tree. After building the tree, it was pruned and smoothing was performed. The optimal value for the minimum number of objects, allowing a new leaf, was determined using five-fold inner-loop cross validation.

#### Double cross validation of kinase-inhibitor interaction models

The predictive ability of models is commonly quantified by the cross-validated squared correlation coefficient, Q^2^. In cross-validation the objects are divided into a number of groups. Models are then developed from the dataset, which has been reduced by one of the groups, and predictions for the excluded objects are calculated. The process is then iteratively repeated until all groups have been omitted once. The Q^2 ^is then calculated as:

where  is the average of the measured outcome values for the N objects in the dataset.

A Q^2 ^> 0.4 is generally considered acceptable for modelling biological data [21]. However, some studies have pointed out that Q^2 ^may give an overly optimistic assessment of model performance in the case that the cross-validation results are used to optimize model parameters or to select the best among many alternative models [49,50]. To remedy this we applied double cross-validation (also called double-loop or nested cross-validation) [51] where the dataset was split into totally 25 parts. In each round of inner cross-validation a model was built on 16/25 of the whole dataset and evaluated on 4/25 of it, while the remaining data were put aside for the outer loop. Once the inner loop cross-validation had found the optimal model, its true performance was verified against 5/25 of data that had never been used during the optimization.

We wanted to evaluate the predictive ability for both new kinase-inhibitor combinations and for new kinases with no measured interaction data. In the former case each part of randomly split dataset comprised about 1/25 of 12,046 kinase-inhibitor pairs and in the latter case it comprised all data for approximately 1/25 of 317 kinases. The squared correlation coefficients from the outer loop of cross-validations for these two different selections are in the following denoted as P^2 ^and P^2^_kin_, respectively (letter P is used instead of Q as in previous studies [51] to emphasize that these are unbiased performance estimates based on external predictions).

## Authors' contributions

ML conceived the study, carried out data analysis, and wrote the initial draft. JESW supervised the study and edited the manuscript. Both authors read and approved the final manuscript.
